# Host–Parasite Relationships in Veterinary Parasitology: Get to Know Your Enemy before Fighting It

**DOI:** 10.3390/ani12040448

**Published:** 2022-02-12

**Authors:** Javier González-Miguel

**Affiliations:** 1 Laboratory of Parasitology, Institute of Natural Resources and Agrobiology of Salamanca (IRNASA-CSIC), C/Cordel de Merinas 40-52, 37008 Salamanca, Spain; jglez@usal.es or javier.gonzalez@irnasa.csic.es; 2 Molecular Parasitology Laboratory, Center of One Health (COH) and Ryan Institute, School of Natural Science, National University of Ireland Galway, H91 DK59 Galway, Ireland

The evolutionary success of parasitism is directly related to the huge number of species that have evolved this way of life. Such success could be explained by the mechanisms evolved by these species to confront and evade the responses of their hosts, together with their capacity to adapt the metabolic processes of their hosts for their own benefit [1]. In regards to veterinary parasitology, these adaptations may lead to chronic infections that cause disease in animals, resulting in loss of production, suffering and even death. In addition, due to the zoonotic potential of a considerable number of parasites, many parasitic diseases of veterinary importance are currently considered as important public health problems. The global market for antiparasitic drugs and chemicals has been estimated at more than USD 10 billion annually for livestock and companion animals [2]. Moreover, the development of drug resistance in protozoan and helminth parasites of veterinary interest has been increasingly reported. The substitution of drugs by other means (e.g., vaccination) for the control of parasitic diseases in animals would have an important impact in the improvement of the quality and food safety, since the recombinant vaccines do not leave residues in food of animal origin. Regrettably and despite their worldwide relevance, the vaccination approaches against parasites carried out so far have not given the expected results. It has been widely suggested that the lack of knowledge regarding the underlying biological, biochemical and immunological components from the host-parasite interface represents one of the main reasons that could explain that currently not many targets have been successfully developed into vaccines against parasites [3]. Consequently, the main objective of this Special Issue is to shed light on the cross-talk interactions between parasites of veterinary importance and their hosts as a fundamental step for the future development of more effective vaccines or for the characterization of new therapeutic targets for the control of these parasitic diseases.

Seven contributions have been published within the Special Issue. These are research papers in which different strategies to unravel host–parasite interactions are carried out. In the first one, Piegari et al. [4] studied the correlation between the infection by the trematode parasite *Dicrocoelium dendriticum* in sheep and the host response in regards to macroscopic lesions, the immunopathological response and histological changes showed by the affected tissues. Despite its high prevalence values in some parts of the world, especially in Europe, and its demonstrated relation to high economic losses in the livestock industry, dicrocoeliosis has received little research attention. The results shown by this manuscript will help to better understand the liver immune response in naturally infected sheep with *D. dendriticum*, as well as opening up the possibility of using the fecal egg count technique as an indirect index of liver damage in dicrocoeliosis. Similarly, Ptáček et al. [5] explored whether some nutritional status characteristics (e.g., low live weight, body-condition score, back muscle or back-fat reserves) could create the suspicion of goats infected with gastrointestinal parasites (specifically, nematodes from the superfamily Trichostrongyloidea and coccidia of the genus *Eimeria*).

Following the line of parasitic diseases that cause a great impact on livestock production, Diosdado et al. [6] studied host–parasite relationships in porcine ascariosis by *Ascaris suum*. Infective larvae of this parasite hatch from ingested eggs in the host small intestine and, from that point, undergo an extensive migration through the host bloodstream that ends in the same location. For that reason, authors hypothesized that these larvae could interact with the components of the haemostatic system of their host for their own benefit. In fact, the published manuscript demonstrated for the first time the ability of *A. suum* larvae to inhibit the coagulation cascade of the host, which could be considered as a mechanism facilitating parasite survival, migration and colonization.

The remaining four publications are focused on the field of host–parasite relationships in fasciolosis. In this foodborne zoonotic disease, resistant isolates of the parasite against the drug of choice, triclabendazole, have been increasingly reported in several countries. This fact has encouraged the development of alternative treatment or other control strategies, such as vaccination trials, for which understanding the cross-talk interactions between hosts and parasites is of paramount importance. The study published by Mas-Coma et al. [7] explored the capacity of llamas to participate in the transmission and epidemiology of fasciolosis in South America. The results showed that although eggs shed by these animals might eventually reach the adult stage, their transmission capacity should be considered negligible, and therefore, llamas should not be considered for control initiatives in fasciolosis. On the same continent, Bargues et al. [8] undertook a detailed phenotypic and molecular study to clarify the presence of big-sized fasciolids found in Ecuador and previously identified as *Fasciola gigantica*, whose distribution is restricted to parts of Africa and Asia, apparently. After developing genotyping of these parasites, authors concluded that *F. gigantica* does not appear to be able to colonize the New World. In the next study, Barbour et al. [9] payed their attention in the newly excysted juvenile worms of *F. hepatica*, which are the first parasitic stage found within the vertebrate host. These juvenile parasites invade the host tissues by crossing the small intestine and migrating to the liver by secreting cathepsin proteases, among other mechanisms. The authors studied the physio-biochemical characteristics of four of these enzymes (FhCB1, FhCB2, FhCB3 and FhCL3) showing their wide variety in terms of activity, optimal pH and substrate specificity, which could be beneficial for the parasite as an efficient digestion system. Finally, in the study published by Molina-Hernández et al. [10], these authors evaluated the hepatic lesions in sheep immunized with a partially protective vaccine against *F. hepatica*. They characterized the presence of degenerated adult worms associated with severe granulomatous inflammation in bile ducts from the above-mentioned sheep by immunohistochemistry.

In sum, the articles published in this Special Issue gathered different approaches for the study of host–parasite relationships in veterinary parasitology from the point of view of epidemiology, genetics, veterinary medicine, molecular biology and others. This fact reflects the multi-disciplinary concept that is required to deal with the complexity of parasitic systems. Consequently, veterinary parasitology research needs to be transdisciplinary, under the umbrella of One Health, in order to be able to generate all the knowledge that is needed to identify and select potential vaccine targets in a more rational way. To reach this objective, (1) setting up new models to understand the host–parasite relationship (specially at the early stages of infections); (2) undertaking omics-based analyses to obtain a comprehensive picture of this interaction; and (3) developing post-genomic approaches (e.g., CRISPR-Cas) will be needed to select the most promising candidates to target in attempts to fight animal parasites (Figure 1).

## Figures and Tables

**Figure 1:**
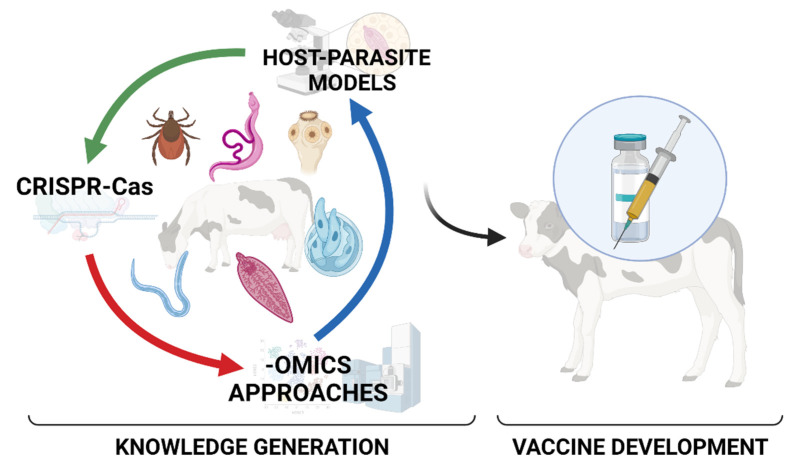
Host–Parasite Relationships in Veterinary Parasitology: Get to Know Your Enemy before Fighting it. Created with BioRender.

## Data Availability

Not Applicable.

## References

[1] Poulin R. (2007). Evolutionary Ecology of Parasites.

[2] Abongwa M., Martin R.J., Robertson A.P. (2017). A Brief Review on the Mode of Action of Antinematodal Drugs. Acta Vet. (Beogr)..

[3] Stutzer C., Richards S.A., Ferreira M., Baron S., Maritz-Olivier C. (2018). Metazoan Parasite Vaccines: Present Status and Future Prospects. Front. Cell. Infect. Microbiol..

[4] Piegari G., Pepe P., De Biase D., d’Aquino I., Bosco A., Cringoli G., Papparella S., Rinaldi L., Paciello O. (2021). Immunopathological Response, Histological Changes, Parasitic Burden, and Egg Output in Sheep Naturally Infected by *Dicrocoelium dendriticum*. Animals.

[5] Ptáček M., Kyriánová I.A., Nápravníková J., Ducháček J., Husák T., Chay-Canul A.J., Zaragoza-Vera C., Cruz-Bacab L., Vadlejch J. (2021). Do Live Weight, Body Condition Score, Back Muscle or Back-Fat Reserves Create the Suspicion of Goats Infected with *Eimeria* or Trichostrongylids?. Animals.

[6] Diosdado A., Simón F., Morchón R., González-Miguel J. (2021). Host-Parasite Relationships in Porcine Ascariosis: Anticoagulant Potential of the Third Larval Stage of *Ascaris suum* as a Possible Survival Mechanism. Animals.

[7] Mas-Coma S., Cafrune M.M., Funatsu I.R., Mangold A.J., Angles R., Buchon P., Fantozzi M.C., Artigas P., Valero M.A., Bargues M.D. (2021). Fascioliasis in Llama, *Lama glama*, in Andean Endemic Areas: Experimental Transmission Capacity by the High Altitude Snail Vector *Galba truncatula* and Epidemiological Analysis of Its Reservoir Role. Animals.

[8] Bargues M.D., Valero M.A., Trueba G.A., Fornasini M., Villavicencio A.F., Guamán R., De Elías-Escribano A., Pérez-Crespo I., Artigas P., Mas-Coma S. (2021). DNA Multi-Marker Genotyping and CIAS Morphometric Phenotyping of *Fasciola gigantica*-Sized Flukes from Ecuador, with an Analysis of the *Radix* Absence in the New World and the Evolutionary Lymnaeid Snail Vector Filter. Animals.

[9] Barbour T., Cwiklinski K., Lalor R., Dalton J.P., De Marco Verissimo C. (2021). The Zoonotic Helminth Parasite *Fasciola hepatica:* Virulence-Associated Cathepsin B and Cathepsin L Cysteine Peptidases Secreted by Infective Newly Excysted Juveniles (NEJ). Animals.

[10] Molina-Hernández V., Ruiz-Campillo M.T., Martínez-Moreno F.J., Buffoni L., Martínez-Moreno Á., Zafra R., Bautista M.J., Escamilla A., Pérez-Caballero R., Pérez J. (2021). A Partially Protective Vaccine for *Fasciola hepatica* Induced Degeneration of Adult Flukes Associated to a Severe Granulomatous Reaction in Sheep. Animals.

